# The New Theory of Yellow Fever Inoculation

**Published:** 1887-07

**Authors:** 


### THE NEW THEORY OF YELLOW FEVER INOCULATION.

On the first day of the meeting of the American Medical Association, at Chicago, upon the call for reports of special committees, Dr. J. McF. Gaston, of Atlanta, as chairman of the committee appointed to memoralize Congress in favor of the bill for investigating the claims of yellow fever inoculation, made a verbal report.

He stated that it was intended to report very briefly what had been done in furtherance of the object to secure a proper consideration for yellow fever inoculation, which was of paramount importance with vaccination against small-pox. The memorial, signed by himself and Dr. Hooper, of Arkansas, did not receive the signature of Dr. Richardson, of Louisiana, the third member of the committee, for reasons satisfactory to himself. It embodied the facts afloded by the experiments of Dr. Domingos Freire, Rio de Janerio; Cremona, of Mexico; Finlay and others who have had opportunities of observing the inocubility of the virus of yellow fever and its protection against the disease in those subjected to its influence.

In the meantime Dr. Joseph Holt, of New Orleans, had brought this matter to the attention of the American Sanitary Association, and had presented a bill to Congress for the appropriation of funds and the appointment of a commission of three physicians to investigate the claims of yellow fever inoculation. Neither his efforts nor the memorial of this committee proved entirely effective, from opposition by those who were interested in the results, and only an insignificant appropriation of $10,000 was made by Congress.

At the second day's session, Dr. Gaston presented the following resolutions as a corrolary to his remarks on the first day:

WHEREAS, An appropriation has been made by Congress for investigating yellow fever inoculation and an eminent bacteriologist has been appointed to examine the data presented in Mexico and Brazil,

*Resolved*, That it is desirable that two other members of the medical profession should be associated in this work, one having practical and clinical acquaintance with yellow fever, and the other being qualified to communicate with the population of the respective localities.

*Resolved*, That a committee of three to be appointed by the President of this Association to communicate this action to President Cleveland, setting forth the grounds for such recommendations.

A reporter of *The Constitution* learned from the doctor upon his arrival from Chicago last evening, that the committee under the resolutions consists of himself as chairman, with Dr. William Brodie, of Detroit, and Dr. Chisolm, of Baltimore. In reply to a question, he stated that a rough draft of a communication to the President had been made by him, a copy of which appears below.

This recommendation looks to the co-operation of two other competent members of the medical profession, with the able microscopist and sanatarian appointed by President Cleveland. It should not, in the opinion of Dr. Gaston, be construed as in any way derogatory to him, nor as being prompted by any opposition to his appointment on the part of the friends of this measure. Dr. Gaston has long since asked that his application for a place on this commission be cancelled and no longer desires an appointment, but is only interested in having the service performed efficiently and done satisfactorily to all concerned, as the original mover in this country.

The following is the memorial:

*To His Excellency Grover Cleveland:*

In compliance with the accompanying resolutions the committee has the honor to state that the appointment of Dr. George Sternberg meets most satisfactorily all the requirements which could be filled by one individual for the prosecution of the important inquiry as to the claims of yellow fever inoculation for the prevention of this disease among our people. Yet it appears to the medical profession that, while the scientific investigation of the data furnished by experimentation is being conducted by this distinguished microscopist, it would be advantageous to have others associated with him who may relieve him of the labor of scrutinizing the statistics and analyzing the evidence afforded by the results of inoculation in Mexico and Brazil. Without any disposition to urge the claims of invividuals to attention, we feel authorized to submit the names of Dr. J. L. Guiteras and Dr. H. M. Lane, the former being familiar with the Spanish and the latter with the Portugese language, and each having a knowledge of the habits and customs of the people in the respective localities. There are many points for the investigation of this commission requiring much care and discrimination in the sifting of testimony which could be accomplished more effectively by three persons than by one or two, and hence the American Medical Association, being desirous of obtaining definite information in regard to the actual results of inoculation as a prophylactic measure, earnestly recommends the addition of two competent members of the medical profession to this commission. It would seem desirable to repeat the application of the cultured virus used by Dr. Domingos Friere, and the deposits from the urine of patients employed by Cremona. The summer of the northern hemisphere being suited to this exploration in Mexico, the present season seems to this committee as appropriate for studying this phase of the inquiry in that country, while in Brazil there is no scope for such observation at this time, but the months corresponding t› our winter afford the climatic conditions favorable to the development of yellow fever which would enable the commission to test the use of inoculation in Rio de Janeiro. It is only by actual experiment with the yellow fever as it prevails in these localities, that the government commission can arrive at a final conclusion as to its efficiency, and the committee trust it may not be transcending the powers entrusted to it to respectfully invite your careful attention to these considerations as good and sufficient reasons for making the addition of two members to your commission. The essential element of microbial origin devolves necessarily upon one acquainted with bacteriology, and skilled in the examination with the miscroscope, as all are ready to allow belong in an eminent degree to Dr. Sternberg. But the conclusion to be reached in regard to the reality in the matter of protection by inoculation, requires familiarity with the language and habits of the people subjected to this scrutiny and practical discrimination as to the nature of the prevailing disease, with rigid observation of all the rules in taking testimony that shall rule out any deception. In these special departments, Drs. Guiteras and Lane have the requisite qualifications. The great desideratum in all experimental developments is to get at the facts, and if it is made to appear that yellow fever may be prevented entirely, or so far modified as to rid it of its terrors by inoculation with the cultivated virus, there can be no longer any doubt of its efficacy. There are so manv conditions involved in the verification of experiments among different classes of people as to necessitate the varied process of investigation contemplated by making additional appointments on the commission.

On being asked when this would be sent to President Cleveland, Dr. Gaston replied that subsequent proceedings precluded its presentation, but that the publication of it would enable those who had voted for the resolutions to understand the intention of the mover, and at the same time convince those who were instrumental in rescinding them, that an important measure has been thwarted by their interference, in support of the past action of the government officials, and for the protection of Sternberg in his work.

Soon after the opening of the Association on Friday morning when few were present, a resolution rescinding the previous action was passed by snap judgment.

In connection with this and in the form of a preamble, Dr. J. B. Hamilton, Surgeon General of the marine service, offered a resolution, that Congress be requested to publish for distribution to the profession a number of copies of the report to be published by Dr. G. M. Sternberg, U. S. A., relating to his researches into the cases of yellow fever in Mexico and Brazil.

Dr. Gaston moved that the two be considered separately, as all were prepared to indorse Dr. Sternberg as the right man in the right place for the bacteriological investigation intrusted to this commission, but his motion, though seconded, was overruled by a call for the previous question, and thus by parliamentary tactics the question was decided in opposition to the voice of a large majority of the Association expressed in the full meeting of the previous day, by the votes upon the motions of Dr. Rohe.

The reporter then asked what was the status as to yellow fever inoculation since this agitation of the matter before the Association, and Dr. Gaston replied that he considered it had received a great impetus in the right direction by bringing the medical men to reflect upon the most effectual means of getting at the facts connected with the experiments made in Mexico and in Brazil, and that the mode in which the final action was taken opened the eyes of many that were heretofore ignorant of the fact that there is a power behind the throne greater than the throne.—*Atlanta Constitution.*

